# Associations of Supermarket Characteristics with Weight Status and Body Fat: A Multilevel Analysis of Individuals within Supermarkets (RECORD Study)

**DOI:** 10.1371/journal.pone.0032908

**Published:** 2012-04-04

**Authors:** Basile Chaix, Kathy Bean, Mark Daniel, Shannon N. Zenk, Yan Kestens, Hélène Charreire, Cinira Leal, Frédérique Thomas, Noëlla Karusisi, Christiane Weber, Jean-Michel Oppert, Chantal Simon, Juan Merlo, Bruno Pannier

**Affiliations:** 1 Inserm, U707, Paris, France; 2 Université Pierre et Marie Curie-Paris6, UMR-S 707, Paris, France; 3 Centre d'Investigations Préventives et Cliniques, Paris, France; 4 Social Epidemiology and Evaluation Research Unit, Sansom Institute for Health Research, University of South Australia, Adelaide, South Australia, Australia; 5 Department of Medicine, St Vincent's Hospital, The University of Melbourne, Fitzroy Victoria, Australia; 6 Department of Health Systems Science, University of Illinois at Chicago, Chicago, Illinois, United States of America; 7 Social and Preventive Medicine Department, Université de Montréal, Montreal, Canada; 8 Université Paris-Est, Lab'Urba - Institut d'Urbanisme de Paris, Créteil, France; 9 EHESP School of Public Health, Rennes, France; 10 ERL7230 CNRS Image, Ville, Environnement, Strasbourg University, Strasbourg, France; 11 Inserm U557, Inra U1125, CNAM, EA3200, University Paris13, Bobigny, France; 12 Department of Nutrition, Pitie-Salpetriere Hospital (AP-HP), CNRH IdF, University Pierre et Marie Curie-Paris6, Paris, France; 13 Lyon University, Inserm U870, Inra U1235, CRNH Rhône-Alpes, Pierre Bénite, France; 14 Unit for Social Epidemiology, Clinical Research Center, Faculty of Medicine, Lund University, Malmö, Sweden; Hospital for Sick Children, Canada

## Abstract

**Purpose:**

Previous research on the influence of the food environment on weight status has often used impersonal measures of the food environment defined for residential neighborhoods, which ignore whether people actually use the food outlets near their residence. To assess whether supermarkets are relevant contexts for interventions, the present study explored between-residential neighborhood and between-supermarket variations in body mass index (BMI) and waist circumference (WC), and investigated associations between brands and characteristics of supermarkets and BMI or WC, after adjustment for individual and residential neighborhood characteristics.

**Methods:**

Participants in the RECORD Cohort Study (Paris Region, France, 2007–2008) were surveyed on the supermarket (brand and exact location) where they conducted their food shopping. Overall, 7 131 participants shopped in 1 097 different supermarkets. Cross-classified multilevel linear models were estimated for BMI and WC.

**Results:**

Just 11.4% of participants shopped for food primarily within their residential neighborhood. After accounting for participants' residential neighborhood, people shopping in the same supermarket had a more comparable BMI and WC than participants shopping in different supermarkets. After adjustment for individual and residential neighborhood characteristics, participants shopping in specific supermarket brands, in hard discount supermarkets (especially if they had a low education), and in supermarkets whose catchment area comprised low educated residents had a higher BMI/WC.

**Conclusion:**

A public health strategy to reduce excess weight may be to intervene on specific supermarkets to change food purchasing behavior, as supermarkets are where dietary preferences are materialized into definite purchased foods.

## Introduction

In recent literature, there has been a considerable interest in the influence that local food environments have on weight status [1]. Because of the mixed evidence and inconsistent findings of previous literature on the food environment and weight status, there is growing debate on the strategies to measure the food environment [2], [3], [4]. Previous research on this issue has overwhelmingly used measures of the food environment defined for the residential neighborhood [5], [6], [7], [8], [9] or the geographic work environment [10] (e.g., the road network distance to the closest supermarket or the density of fast-food restaurants).

A major limitation of these measures, however, is that they do not reflect the *personal food environment* of individuals, as they do not account for whether participants effectively use or do not use food outlets located near their residence or workplace. Neighborhood-based measures of the local food environment may be meaningless for people who shop far from their residence. As suggested by studies documenting relationships between the type of store where people shop and dietary behavior [11], a necessary need, therefore, is to derive *personal measures of the food environment* through the identification of the food outlets people effectively use. Moreover, to assess whether supermarkets are appropriate contexts for developing preventive nutritional interventions, it is relevant to explore between-supermarket (in addition to between-residential neighborhood) variations in weight status and abdominal fat through multilevel analysis.

In the RECORD Cohort Study [12], [13], [14], [15], [16], the supermarket where participants did most of their food shopping was assessed with its brand, exact location, and characteristics. The present study examined (i) whether participants who did most of their food shopping in the same supermarket had a more comparable body mass index (BMI) and waist circumference (WC) than participants who shopped in different supermarkets (after accounting for the residential neighborhood); and (ii) whether differences in BMI and WC between participants shopping in different supermarkets were explained by the distance from residence to the supermarket (which may influence food purchasing behavior [17]), the socioeconomic status (SES) of supermarket catchment area (possibly related to food preferences and purchasing power of consumers and types of foods available), the supermarket brand, the supermarket type, supermarket size, and by the quality of fruits and vegetables in the supermarket.

## Methods

### Ethics

The study protocol was approved by the French Data Protection Authority. All the participants signed an informed consent to enter the study.

### Population

The RECORD Cohort Study (“Residential Environment and CORonary heart Disease”, www.record-study.org) includes 7 290 participants who were recruited between March 2007 and February 2008 [12], [13], [14], [15], [16]. Participants were beneficiaries of the French National Health Insurance System for Salaried Workers, which offers a free medical examination every 5 years to all working and retired employees and their families (corresponding to 95% of the population of the Paris Ile-de-France region). Participants were recruited without *a priori* sampling (convenience sample) among the eligible persons attending these 2-hour-long preventive checkups conducted by the Centre d'Investigations Préventives et Cliniques in four of its health centers located in the Paris Ile-de-France region (Paris, Argenteuil, Trappes, and Mantes-la-Jolie). People seen at these preventive health checkups are not unhealthier than the average population, and attend these health centers either on a voluntary basis, through the referral of their family physician or workplace physician, following the advice of peers, etc. Eligibility criteria were as follows: age 30 to 79 years; ability to complete study questionnaires; and residence in one of the 10 (out of 20) administrative districts of Paris or 111 other municipalities of the metropolitan area selected *a priori*. These municipalities and districts were chosen so as to reflect disparities between advantaged and disadvantaged territories (with overrepresentation of disadvantaged municipalities) and disparities between urban and suburban areas. Among people presenting at the health centers who were eligible based on age and residence, 10.9% were not selected for participation because of linguistic or cognitive difficulties in filling out study questionnaires. Of the persons selected for participation, 83.6% accepted to participate and completed the entire data collection protocol.

Participants were geocoded with accuracy based on their residential address in 2007–2008. Research assistants rectified all incorrect or incomplete addresses with the participants by telephone. Extensive investigations with local Departments of Urbanism were conducted to complete the geocoding. Precise spatial coordinates and block group codes were identified for 100% of the participants.

Due to missing information on the supermarket (see below) or in anthropometric measures, the final sample available for analysis of BMI and WC comprised 7 072 and 6 926 participants, respectively.

### Measures

Summary information on the scales of analysis and scales of measurement of the variables is provided in Table 1. Details on the anthropometric measures (body mass index and waist circumference) and individual sociodemographic variables analyzed in the present study are provided in Table 2.

**Table 1:** Summary of the scales considered in the analyses (hierarchical modeling) and in the measurement of the variables^a^.

	Individual level	Residential neighborhood level	Supermarket level
Scales of analysis	Individual statistical units	Administrative neighborhoods, TRIRIS areas (random effect)	Primary supermarket (random effect)
Scales of measurement of the variables	Body mass index and waist circumferenceIndividual sociodemographic variables*Individualized* neighborhood socioeconomic status *Individualized* distance to the supermarket		Supermarket type and brandSupermarket size Supermarket area socioeconomic statusQuality of fruits and vegetables (*supermarket-level aggregate of individual variables*)

a The analytic scales refer to the levels accounted for in the multilevel models, while the measurement scales refer to the levels of definition of the different variables. Expressions in italic indicate (i) that exposure to neighborhood socioeconomic conditions was assessed at the individual level within circular areas centered on residences; (ii) that the supermarket-level variable on the quality of fruits and vegetables was based on the aggregation of individual perceptions; and (iii) that the distance to the supermarket was assessed at the individual level.

**Table 2:** Description of the individual-level, residential neighborhood-level and supermarket-level variables analyzed, RECORD Cohort Study, Paris Metropolitan Area, 2007–2008.

Variable	Description
Individual anthropometry	
Body mass index (kg/m^2^)	Determined from height (measured using a wall-mounted stadiometer) and weight (measured using calibrated scales) recorded by a nurse [18]
Waist circumference (cm)	Measured using an inelastic tape placed mid way between the lower ribs and iliac crests on the mid-axillary line, with the subjects standing [19]
Individual sociodemographic variables	
Age	Specified both as a linear and a quadratic term
Marital status	2 classes: living alone or as a couple
Individual education	4 classes: no education; primary education and lower secondary education; upper secondary education and lower tertiary education; upper tertiary education
Mother's education	3 classes: primary school or less; secondary school; tertiary school
Father's education	3 classes: primary school or less; secondary school; tertiary school
Occupation	4 categories: blue collar workers; low white collar workers; intermediate occupations; high white collar workers
Employment status	3 categories: employed; unemployed; retired
Household income	Adjusted for household size and divided into 4 categories based on the quartiles
Self-reported financial strain	Yes or no
Dwelling ownership	Yes or no
Human development of countryof birth	2004 Human Development Index [20] of country of birth; 4 categories: born in mainland France, and in countries with a high level, an intermediate level, and a low level of human development [21]
Residential neighborhood and supermarket neighborhood variables computed on different scales^a^	
Neighborhood education	Proportion of residents aged 15 or over with a tertiary education (2006 Census) in 4 categories
Neighborhood median income	Median income in 2006 (Tax Registry of DGI, General Directorate of Taxation) in 4 categories
Neighborhood dwelling values	Mean value of dwellings sold in 2003–2007 (Paris-Notaries) in 4 categories
Variables related to the participants' primary supermarket	
Supermarket brand	Binary variable for each store brand in which at least 30 participants were shopping (corresponding to 7 006 of the 7 131 participants, who were shopping in 17 different supermarket chains); other supermarket brands were all combined in a pooled category
Supermarket type	Citymarkets, hypermarkets, small/large supermarkets, hard discount supermarkets, organic shops (see Information S1 for additional information)
Supermarket within one's residential neighborhood	Binary variable: one's supermarket located in or out of one's administrative neighborhood
Distance to the supermarket	Street network distance from residence to one's supermarket (ArcInfo 10 Network Analyst and street network data from the National Geographic Institute)
One's supermarket as the closest available	Calculation of street network distance to the closest supermarkets (2007 Trade Dimension database with all available supermarkets geocoded). One's supermarket:as the closest (binary variable)- as the closest of a particular type: citymarket, small/large supermarket, hypermarket, hard discount supermarket (binary variable)- as the closest of a particular brand (binary variable)
Supermarket size^b^	Information on each participant's supermarket retrieved from the Trade Dimension database through the supermarket business identification code:- Supermarket area (m^2^)- Number of cash desks- Number of employees
Quality of fruits/vegetables in one's supermarket	Supermarket-level random effect of a two-level multilevel logistic model in which participants' rating of the quality of fruits/vegetables in their supermarket was taken as the outcome (ecometric approach [22], [23], [24])

a Variables were computed on different scales, with a circular radius from 100 to 10 000 m. Socioeconomic status of supermarket catchment areas was also determined within circular areas with a varying radius corresponding to the 75^th^ percentile of the straight-line distance from home to the participants' primary supermarkets of each particular brand (brand-specific scales).

b These variables were missing for the supermarkets utilized by 6.5% of the participants.

#### Collective entities: residential neighborhoods and supermarkets

Participants resided in 1 910 different census block group areas. The median number of residents in these local units was 2 425 (interquartile range: 2 083, 2 932) in the 2006 Population Census.

The participants were asked to report the brand and address of the supermarket where they did most of their food shopping (the survey question referred to supermarkets rather than food stores in general because, as shown with the data provided below, most people in France are shopping in chain supermarkets). During face-to-face contact, trained survey technicians sought to collect as much information as possible to identify these supermarkets. During the baseline survey, technicians did not, however, perform online searches to assist participants who were unable to identify their supermarket.

Each participant's primary supermarket could not always be straightforwardly identified. For example, Franprix is a brand of small supermarkets, and in Paris, there are often many Franprix very close to each other. Also, in certain cases, there were more than one supermarket of the same brand in the same street. In these cases and others, participants were systematically telephoned in the months subsequent to their health examination, in attempts to precisely identify the supermarket where they shopped (790 participants were so contacted). Technicians conducting these calls used Google Maps and the websites of the different supermarket brands to assist them in their searches. In the end, the official business identification code (SIRET) of each supermarket was retrieved.

Among the 7 290 participants, three participants who reported doing most of their shopping at the market (i.e., comprising numerous independent shopkeepers), 26 participants who did most of their food shopping via the internet, and 130 participants who did not make most of their shopping in a supermarket or could not provide enough information to identify the supermarket were removed from the study. For five participants who could not choose which one of two supermarkets was their main source of food, the largest supermarket was used for analysis. After the exclusions listed above, 7 131 participants were coded as conducting most of their food shopping in 1 097 distinct supermarkets.

#### Residential neighborhood- and supermarket-level contextual variables

Three distinct characteristics were considered for residential neighborhood SES and SES of supermarket catchment area (hereafter referred to as supermarket neighborhood SES) (see Table 2). Using databases geocoded at the building level, these characteristics were determined within circular areas of different scales centered on the exact place of residence or on the exact supermarket location [25], [26]. Moreover, to account for the fact that supermarket catchment areas greatly vary in size between supermarket brands, SES of supermarket catchment areas was also determined within circular areas with brand-specific radiuses (see Table 2).

Variables related to each participant's supermarket are described in Table 2: supermarket brand (Table 3 provides descriptive information on each brand), supermarket type (see Information S1 for additional information on supermarket types), whether one's supermarket is within one's residential neighborhood, the distance to the supermarket, whether one's supermarket is the closest available, supermarket size, and the quality of fruits/vegetables in one's supermarket.

**Table 3:** Characteristics of the main supermarket brands and adjusted associations between supermarket brands and BMI or WC (brands are ranked by supermarket type and alphabetical order), RECORD Cohort Study, Paris Metropolitan Area, 2007–2008.

Brand name	Type	Size (m^2^)^a^	75^th^ perc. of distance (m)^b^	Number of participants	Δ BMI^c^ in kg/m^2^	95% CI	Δ WC^c^ in cm	95% CI
Monoprix	Citymarket	2 337	925	1 106	Ref.		Ref.	
Champion	Small/large supermarket	1 819	3 462	632	+0.4	−0.0, +0.8	+1.4	+0.3, +2.5
Franprix	Small/large supermarket	575	870	931	+0.4	+0.0, +0.7	+1.3	+0.3, +2.2
G20	Small/large supermarket	391	1 343	85	+0.4	−0.5, +1.3	+1.3	−1.1, +3.8
Intermarché	Small/large supermarket	1 420	12 428	155	+0.3	−0.4, +1.0	+0.4	−1.5, +2.2
Shopi	Small/large supermarket	519	720	61	+0.1	−0.9, +1.1	−0.1	−2.9, +2.9
Simply Market	Small/large supermarket	1 506	2 228	290	+0.4	−0.1, +0.9	+1.2	−0.3, +2.6
Casino	Small/large supermarket & hypermarket	3 000	942	273	+0.6	+0.1, +1.2	+2.0	+0.5, +3.5
Système U	Small/large supermarket & hypermarket	1 346	4 866	170	+0.3	−0.3, +1.0	+2.1	+0.3, +3.9
Auchan	Hypermarket	12 010	4 241	752	+0.4	−0.1, +0.8	+1.2	−0.1, +2.4
Carrefour	Hypermarket	11 904	4 639	1 135	+0.5	+0.1, +0.9	+1.5	+0.4, +2.6
Cora	Hypermarket	8 992	2 503	45	+1.6	+0.4, +2.8	+3.5	+0.1, +6.8
Leclerc	Hypermarket	4 997	5 714	559	+0.4	−0.0, +0.8	+1.3	+0.1, +2.5
Aldi	Hard discount	635	3 057	38	+0.8	−0.5, +2.2	+2.4	−1.2, +5.9
Ed	Hard discount	476	1 683	364	+0.6	+0.1, +1.1	+2.3	+1.0, +3.7
Leader Price	Hard discount	859	2 030	248	+0.6	+0.1, +1.2	+1.6	+0.1, +3.2
Lidl	Hard discount	635	3 031	109	+1.2	+0.4, +2.0	+3.6	+1.4, +5.8

Abbreviations: BMI, body mass index; CI, confidence interval; WC, waist circumference.

a Mean size of the supermarkets of each specific brand weighted by the number of participants using each supermarket.

b Street-network distance from home to the participants' supermarkets.

c Associations were estimated from cross-classified multilevel linear models adjusted for individual sociodemographic characteristics, neighborhood education, and distance to the supermarket. They were not adjusted for supermarket neighborhood education, as supermarket neighborhood SES was hypothesized to mediate the possible influence of brands.

### Statistical analysis

After deriving descriptive statistics, we estimated multilevel linear/logistic models with individual education, household income, neighborhood education, and neighborhood income as explanatory factors, and the following variables as the outcomes: (i) one's primary supermarket located or not in one's administrative residential neighborhood; (ii) street network distance to one's primary supermarket; (iii) shopping or not at the closest available supermarket; (iv) shopping or not at the closest supermarket of a particular type (citymarket, small/large supermarket, hypermarket, hard discount supermarket); (v) shopping or not at the closest supermarket of a particular brand.

For BMI and WC separately, we estimated cross-classified multilevel linear models [27] with two distinct random effects at the neighborhood level and at the supermarket level (levels not nested within each other).

Models adjusted for age and sex were first estimated. A second series of models adjusted for individual sociodemographic variables assessed the spatial scale on which residential and supermarket neighborhood SES were most strongly associated with BMI and WC [25], [26]. The different supermarket variables were then included in a third series of models adjusted for individual and residential neighborhood SES. At each step, only those variables that remained associated with the outcomes were retained. We assessed interactions between the coefficients for individual or residential neighborhood SES and supermarket characteristics.

All models were estimated with SAS 9.2. The Akaike Information Criterion (AIC) was used to compare the models: the lower the AIC, the better the overall combination of fit to the data and parsimony of the model.

## Results

Descriptive data on the study sample are provided in Information S2.

### Descriptive data on participants' shopping behavior

Just 11.4% of participants reported shopping for food primarily within their residential census block group neighborhood. Even on a larger scale, no more than 64.2% of the participants did most of their food shopping in their municipality of residence. Regarding street network distance to the primary supermarket, as much as 50% and 31% of the participants had their primary food store located further than 1 km away and 2 km away, respectively, from their residence.

The proportion of participants who shopped in their administrative residential neighborhood tended to increase with socioeconomic status, especially with education of neighborhood residents (Information S3). On the opposite, descriptive data and regression modeling indicated that the average street network distance to the supermarket was larger for residents of high education neighborhoods (Information S3). The contradiction is only apparent: residents of high education neighborhoods more frequently shopped in their residential neighborhood (possibly due to more frequently residing in urban centers) but at the same time covered larger average distances to their primary supermarket (some of them, e.g., having holiday homes or working far from their residence).

Comparing the effective participants' primary supermarket to the available supermarkets nearby their residence, we found that 29.5% of the participants performed most of their food shopping at the closest available supermarket. As shown in Information S3, participants residing in neighborhoods with a high average education level were more likely to shop at the closest supermarket available from their residence.

### Models for BMI and WC

The mean BMI of participants was 25.5 kg/m^2^ (interdecile range: 20.7, 30.7). Mean WC was 77.7 cm (interdecile range: 65, 94) among women and 89.0 cm (interdecile range: 76, 103) among men.

After adjustment for age and sex, models that included both residential neighborhood-level and supermarket-level random effects had a better combination of fit to the data and parsimony than models including only the residential neighborhood- or the supermarket-level random effect (with a 18.2 and 17.4 points lower AIC, respectively, for BMI and a 15.0 and 9.4 points lower AIC for WC).

In models including both residential neighborhood-level and supermarket-level random effects, there were variations in BMI and WC both between residential neighborhoods and between supermarkets (see Information S4). Even after accounting for each person's residential environment, participants who shopped in the same supermarket had a more comparable BMI or WC than those shopping in different supermarkets. Overall, 4.2% and 3.1% of the variations in BMI and WC were at the residential neighborhood level, and 2.5% and 2.2% of the variations were at the supermarket level.

Individual socioeconomic variables associated with BMI or WC are reported in Information S5. As previously reported [14], after individual-level adjustment, residential neighborhood education was more strongly associated with BMI and WC than were neighborhood income or neighborhood dwelling values. BMI and WC increased with decreasing neighborhood education. As shown in Information S6, model fit was the best when residential neighborhood education was assessed within 500 m radius circular areas.

Similarly, after adjustment for individual characteristics, the relationship between supermarket neighborhood SES and BMI or WC was stronger when assessed with education than with income or dwelling values. As shown in Information S6, model fit was the best when supermarket neighborhood education was assessed within 5 000 m radius circular areas centered on the supermarket, i.e., on a much broader scale than residential neighborhood education. Supermarket neighborhood education assessed within brand-specific radius circular areas led to a slightly better fit to the data only in the model for BMI. Accordingly, supermarket neighborhood education was assessed in circular areas with brand-specific radiuses in the model for BMI and in 5 000 m radius circular areas in the model for WC.

There was a strong correlation between residential neighborhood education and supermarket neighborhood education. Nonetheless, when residential neighborhood education and supermarket neighborhood education were introduced together into the models, the two variables remained associated with either BMI or WC (Table 4).

**Table 4:** Associations^a^ between residential neighborhood education, supermarket type, distance to the supermarket, and supermarket neighborhood education and BMI or WC, RECORD Cohort Study, Paris Metropolitan Area, 2007–2008.

	Δ BMI in kg/m^2^	95% CI	Δ WC in cm	95% CI
Residential neighborhood education				
High	Ref.		Ref.	
Medium-high	+0.1	−0.2, +0.4	+0.1	−0.7, +0.8
Medium-low	+0.2	−0.2, +0.5	+0.1	−0.8, +0.9
Low	+0.8	+0.4, +1.3	+2.0	+1.0, +3.0
Supermarket type				
Citymarkets	Ref.		Ref.	
Small/large supermarkets	+0.3	+0.0, +0.6	+1.2	+0.4, +2.0
Hypermarkets	+0.4	+0.0, +0.7	+1.2	+0.3, +2.2
Hard discount supermarkets	+0.7	+0.3, +1.1	+2.2	+1.1, +3.3
Organic shops	−2.1	−3.4, −0.9	−6.1	−9.4, −2.8
Distance to the supermarket				
Short	Ref.		Ref.	
Medium-short	+0.1	−0.2, +0.4	+0.2	−0.5, +1.0
Medium-long	+0.0	−0.3, +0.3	+0.4	−0.4, +1.2
Long	+0.3	−0.0, +0.6	+1.1	+0.2, +2.0
Supermarket neighborhood education^b^				
High	Ref.		Ref.	
Medium-high	+0.2	−0.2, +0.5	+0.6	−0.2, +1.4
Medium-low	+0.1	−0.2, +0.5	+0.8	−0.1, +1.6
Low	+0.5	+0.1, +0.9	+1.0	+0.0, +2.1

Abbreviations: BMI, body mass index; CI, confidence interval; WC, waist circumference.

a The following variables were simultaneously introduced into the models: individual sociodemographic characteristics, residential neighborhood education, supermarket type, distance to the supermarket, and supermarket neighborhood education.

b Based on the AIC, supermarket neighborhood education was measured in brand-specific radius areas in the model for BMI and in 5 000 m radius areas in the model for WC.

After full adjustment for individual, residential neighborhood, and supermarket characteristics, participants who did most of their food shopping far from home had a slightly larger WC and tended to have a slightly higher BMI (Table 4). After adjustment, the quality of fruits and vegetables in the supermarket and supermarket size showed no association with BMI or WC.

After controlling for individual and residential neighborhood SES and distance to the supermarket, and using the Monoprix brand (expensive citymarkets located in city centers) as the referent, participants shopping in certain supermarket brands, especially in hypermarkets such as CORA or in hard discount supermarkets such as Ed or Lidl, had a greater BMI and WC (Table 3).

Considering supermarket type instead of supermarket brands, people shopping in small/large supermarkets, hypermarkets, and more particularly in hard discount supermarkets had a greater BMI and WC compared to those shopping in citymarkets, while participants shopping in organic shops had a markedly lower BMI and WC (Table 4).

**Figure 1:**
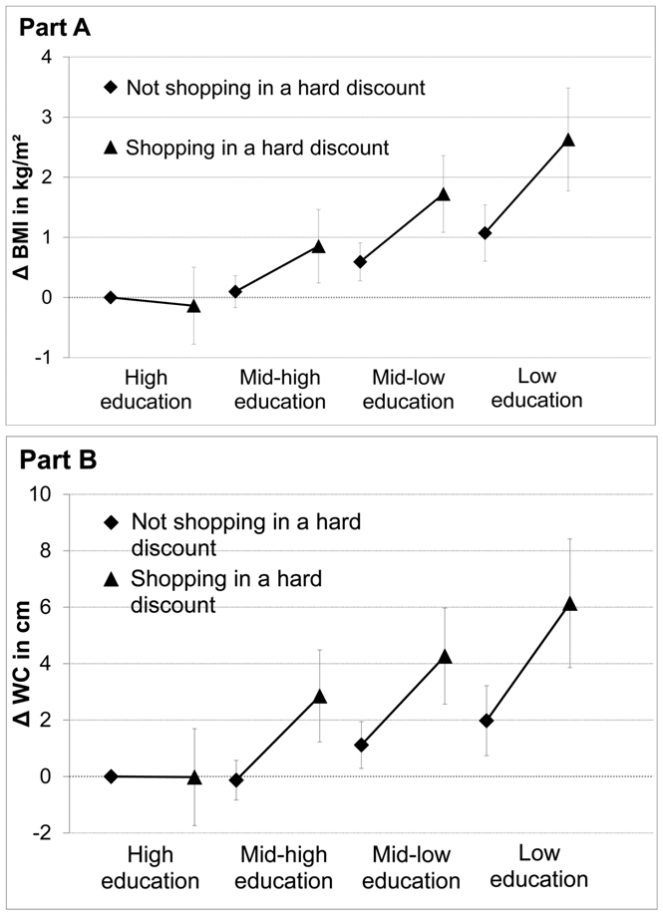
Interactions between effects of individual education and shopping in hard discounts on the anthropometric variables. The interactions were estimated from cross-classified multilevel linear models for body mass index (BMI) (part A) and waist circumference (WC) (part B) adjusted for individual sociodemographic characteristics, residential neighborhood education, supermarket type, distance to the supermarket, and supermarket neighborhood education (interactions assessed with a variable combining categories of the two variables). RECORD Cohort Study, Paris Metropolitan Area, 2007–2008.

There was a strong interaction between the coefficients for individual education and shopping in a hard discount supermarket. Models for BMI and WC re-estimated with a variable combining individual education and shopping in a hard discount store (Figure 1) indicated that the association between shopping in a hard discount store and BMI or WC was markedly stronger for lower education levels.

As detailed in Information S7, relationships between supermarket type and BMI/WC were re-estimated after propensity score matching, i.e., among participants shopping in different types of supermarkets matched according to their probability to shop in a specific supermarket type (probability estimated in function of a number of individual socioeconomic characteristics). In these analyses, as shown in Table 5, participants shopping in small/large supermarkets and hypermarkets tended to have a higher BMI/WC than those shopping in citymarkets. However, relationships between shopping in hard discount supermarkets and anthropometry were lost in these propensity score matched analyses, probably due to the sharp decrease in sample size after matching (which decrease resulted from a limited exchangeability of participants shopping in citymarkets and hard discount supermarkets according to their individual socioeconomic profile).

**Table 5:** Associations^a^ between supermarket type and BMI or WC before and after matching on the propensity of exposure, RECORD Cohort Study, Paris Metropolitan Area, 2007–2008.

	Before matching		After matching	
	Coefficient	95% CI	Coefficient	95% CI
Models for BMI (in kg/m^2^)				
Small/large supermarket vs. citymarket	+0.44	+0.17, +0.72	+0.25	−0.12, +0.62
Hypermarket vs. citymarket	+1.02	+0.68, +1.36	+0.74	+0.03, +1.46
Hard discount vs. citymarket	+0.96	+0.55, +1.37	+0.39	−0.44, +1.21
Models for WC (in cm)				
Small/large supermarket vs. citymarket	+1.47	+0.69, +2.25	+1.18	−0.03, +2.39
Hypermarket vs. citymarket	+2.78	+1.92, +3.66	+2.72	+0.85, +4.59
Hard discount vs. citymarket	+2.75	+1.62, +3.88	+0.91	−1.06, +2.87

Abbreviations: BMI, body mass index; CI, confidence interval; WC, waist circumference.

a The models were adjusted for individual sociodemographic characteristics. Due to the low sample sizes after matching, the models reported here (before and after matching) are not adjusted for neighborhood education or other supermarket variables.

Supplementary analyses also indicated that all of the associations between supermarket characteristics and WC disappeared after adjustment for BMI [28].

## Discussion

The results indicate that a greater distance from one's home to one's primary supermarket, a lower SES of supermarket catchment area, and shopping in specific supermarket brands or supermarket types (especially in hard discount supermarkets among low educated individuals) were associated with a greater BMI and WC. This research supports the utility of deriving personal measures of the food environment by geocoding supermarkets where people do most of their food shopping and the relevance of multilevel studies of individuals nested within food stores for targeting nutritional interventions to specific supermarkets showing higher obesity prevalence [29].

### Strengths and limitations

Strengths of the present study include (i) the time-consuming and meticulous geocoding of participants' supermarkets, (ii) the assessment of abdominal fat beyond general weight status, (iii) the cross-classified multilevel regression models [27] estimated to simultaneously partition variance in BMI and WC between residential neighborhoods and supermarkets, (iv) the propensity score matching analyses performed to examine whether supermarket effects are separable from individual socioeconomic effects, and (v) the investigation of spatial scale of predictors that revealed that supermarket neighborhood education was related to BMI/WC on a much broader scale than residential neighborhood education.

Study limitations include (i) the cross-sectional nature of the data that did not allow us to investigate whether frequenting specific supermarkets is related to weight gain over time, (ii) the inability to control for food preferences that condition supermarket choice and therefore likely confound the relationships of interest (discussed below), (iii) the limited amount of information available on each supermarket [30], e.g., the absence of data on the proportion of shelf space devoted to each type of food [31] as more direct exposures than those measured in the present study, and (iv) the absence of information on participants' food purchasing behavior as a mediator of the supermarket–body weight relationship.

### Main study findings

Most previous studies of the relationship between the food environment and weight status have relied on impersonal measures of the food environment related to residential or geographic work environments assumed to reflect where individuals obtain food [5], [6], [7], [8], [9], [10]. However, the present data remarkably indicate that as much as 88.6% and 35.8% of participants did not shop in their own neighborhood or municipality, respectively. Therefore, neighborhood-based measures of the food environment may in many circumstances provide incomplete picture of individuals' food environments. Moreover, our data suggest that a focus on the residential neighborhood may differentially affect the quality of measurement of the food environment in the different socioeconomic groups, thereby potentially biasing the assessment of the role of the food environment in socioeconomic disparities in obesity risk and poor health. As a complementary strategy, there is a need to develop personal measures of the food environment based on participants' frequenting of given supermarkets or food providers. Such a strategy enabled (i) accounting for food shopping supermarkets, in addition to residential neighborhoods, as a component of group-level variability in multilevel analyses, and (ii) deriving supermarket-level variables to assess in relation to BMI and WC.

Longer distances between one's home and primary supermarket tended to be related to greater BMI and WC, as also reported previously [17]. People shopping far from their residence may less frequently go to their supermarket and may accordingly rely on less fresh products. It is not clear, however, whether this process may contribute to the relationship between distance to the supermarket and weight status.

**Figure 2:**
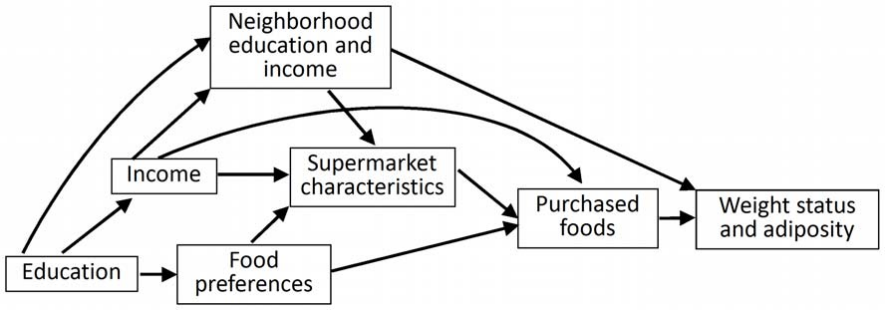
Directed acyclic graph describing the hypothesized relationships.

Regarding supermarket characteristics, independent associations were documented between, on the one hand, supermarket brand, supermarket type, and educational level of supermarket catchment area and, on the other hand, BMI and WC. Even if the model was adjusted for individual and neighborhood variables that may condition supermarket choice, associations reported here reflect a mixture of causal effect and residual confounding that are intrinsically difficult or almost impossible to disentangle (see Figure 2). Of course, our discussion of different causal scenarios does not imply that we draw any causal conclusion from our observational data.

Causal effects of supermarket brand, type, and SES (in support of which our observational data do not provide solid evidence) may stem from the differential availability of healthy foods such as fruits/vegetables or fish, from the availability of low-cost energy-dense foods in hard discount supermarkets, or from the differential advertisement, showcasing, or nutritional labeling of these products in the different supermarkets, all of which may constrain or influence individual purchasing behavior [32], [33].

We reviewed the published evidence on the nutritional quality of low priced or hard discount food products as relevant to correctly interpret the associations documented in our study between shopping in hard discount supermarkets and BMI/WC. A study conducted in France reported that 36% of surveyed consumers thought that low priced products are of lower quality than the branded ones [34]. In coherence with this perception, a French study published more than 15 years ago indicated that branded products were more regularly of high quality (but not always better) in terms of nutrient content, food security, and taste indicators than low cost ones [35]. A more recent study indicated that, within a given food category, the lowest-priced foods were not different from the equivalent branded products in terms of overall energy or total fat content [36]. Another French study published in 2009 reported no systematic difference between low priced and branded products in terms of nutrient content, raw materials, microbiological analysis, or taste [37]. However, in the aforementioned French study, a weak relationship suggested that the overall quality of ingredients increased with the price of foods (within a given food category) [36]. Moreover, basic nutritional information and dietary recommendations were less often provided on low priced foods than on branded products [36], [38], [39]. Overall, the published information is scarce and provides only mixed evidence in support of the idea that hard discount supermarkets are obesogenic nutritional environments [38].

As illustrated in Figure 2, the most significant source of bias for the associations between supermarket characteristics and BMI or WC likely stems from confounding from characteristics that are independent determinants of both supermarket choice and nutritional status. There are at least three major confounding mechanisms possibly involved in the reported associations. The first is related to neighborhood SES, the second one to individual/household financial capacities (low income populations are constrained to rely on energy-dense, nutrient-poor foods to obtain daily calories at an affordable cost [40]), and the third one is related to food preferences.

Regarding the first bias, residential neighborhood SES influences which supermarket brands are available in one's local vicinity (SES is an important criterion in geomarketing location strategies [14]). Moreover, neighborhood SES is associated with other exposures influencing weight status (e.g., opportunities for physical activity) [41]. Therefore, neighborhood SES may be a major source of confounding in the associations between supermarket characteristics and weight status or abdominal fat [42].

As shown in Figure 2, confounding by individual financial capacities and food preferences is attributable to the fact that individual income and dietary preferences not only influence supermarket choice, but also shape the food purchasing behavior of typical customers in each supermarket (i.e., typical customers of supermarket A would have a different purchasing behavior than typical customers of supermarket B if they were constrained to shop in supermarket B).

We addressed the biases related to neighborhood SES and financial capacities by controlling for residential neighborhood education and testing adjustment for household income. However, even though the models were adjusted for individual education, we did not control for dietary preferences, which may confound the estimated associations.

Moreover, it is important to keep in mind that propensity score matching analyses revealed that it is difficult to disentangle the supermarket type – BMI/WC associations from the effects of individual socioeconomic factors. For example, there was evidence that adjustment for individual socioeconomic variables of the relationships between shopping in a hard discount supermarket and BMI/WC was to some extent based on excessive models' extrapolations.

In order to address these issues, future studies should combine information on the types and brands of participants' primary supermarkets, on the food products sold in these supermarkets, on individual food purchasing behavior and consumption, and on anthropometric indices.

### Implications

Whether partly attributable to confounding or not, the results of the study have important implications in suggesting that the supermarket where most of the food shopping is done could be targeted by public health interventions aimed at promoting adequate nutritional practices [29]. While numerous studies proposed to consider residential neighborhoods to identify high-priority areas for interventions, far fewer studies have suggested supermarkets as the basic unit for targeting interventions, as implied by the multilevel analysis of individuals nested within supermarkets reported here. Implementing interventions focused on food purchasing behavior in specific supermarkets may be an efficient strategy because supermarkets are the very place where dietary preferences are concretely materialized and translated into a definite set of purchased foods. The present study that considered various supermarket characteristics may prove useful in identifying supermarkets, based on their brand and catchment area characteristics, where nutritional interventions may be particularly beneficial.

## Supporting Information

Information S1 **Additional information on supermarket type and supermarket brands.**
(DOC)

Information S2 **Descriptive information on the study sample.**
(DOC)

Information S3 **Information on participants' shopping behavior.**
(DOC)

Information S4 **Between-neighborhood and between-supermarket variations in BMI and WC from models adjusted for age and gender.**
(DOC)

Information S5 **Models with individual and residential neighborhood variables associated with BMI and WC.**
(DOC)

Information S6 **Identification of the residential and supermarket neighborhood socioeconomic variables most strongly associated with BMI or WC and assessment of the optimal spatial scale of measurement of the variables.**
(DOC)

Information S7 **Relationships between supermarket type and body mass index (BMI) or waist circumference (WC) estimated through propensity score matching.**
(DOC)
