# Supplementary material for: Associations of Supermarket Characteristics with Weight Status and Body Fat: A Multilevel Analysis of Individuals within Supermarkets (RECORD Study)
Source: PLoS One. 2012 Apr 4;7(4):e32908. doi: 10.1371/journal.pone.0032908 (PMC3319546; doi:10.1371/journal.pone.0032908)
Supplement: Information S1 — Additional information on supermarket type and supermarket brands. (DOC) [file pone.0032908.s001.doc]

**Supporting information S1 – Additional information on supermarket type and supermarket brands**

The different supermarket types considered in the analyses include hypermarkets, small/large supermarkets, hard discount supermarkets, citymarkets, and organic shops. We provide here additional information on these supermarket types.

Hypermarkets have a floor space higher than 2 500 m², and generally sell between 50 000 and 80 000 different items. They mainly sell food products, with the share of non-food products increasing with the size of the hypermarket. Auchan, Carrefour, Cora, and Geant Casino are large hypermarkets, while Leclerc and Hyper U are medium size hypermarkets. Hypermarkets are mainly located on the outskirts of cities and in large commercial malls.

Supermarkets are often subdivided into small supermarkets (floor space between 400 and 1 000 m²) and large supermarkets (floor space between 1 000 and 2 500 m²). Small/large supermarkets generally sell between 5 000 and 10 000 different items. Small supermarkets are mainly located in residential neighborhoods and small towns, while large supermarkets are often located on the outskirts of cities. Small/large supermarkets are usually quite expensive.

In hard discount supermarkets, floor space is generally comprised between 300 and 1 500 m² (average = 600 m²). Hard discount supermarkets generally sell between 700 and 1 000 different items. They are often located on the outskirts of cities or in residential neighborhoods, but are now increasingly found in city centers. There are differences between hard discount brands. For example, Aldi does not sell products of recognized brands, whereas Ed and Lidl do. Lidl is described as the least expensive hard discount brand in France. Leader Price generally sells more items than the other hard discount brands.

Nowadays, citymarkets are only represented by the Monoprix brand. With an average floor space of 1 500 m², citymarkets usually sell between 5 000 and 10 000 different items. The proportion of non-food products sold in citymarkets is larger than that sold in supermarkets of comparable size. Citymarkets are often located in city centers.

Organic shops as a supermarket type were defined by aggregating the following brands: “Les Nouveaux Robinson”, “Naturalia”, “Un monde Bio”, and “Biocoop”.
