# Supplementary material for: Associations of Supermarket Characteristics with Weight Status and Body Fat: A Multilevel Analysis of Individuals within Supermarkets (RECORD Study)
Source: PLoS One. 2012 Apr 4;7(4):e32908. doi: 10.1371/journal.pone.0032908 (PMC3319546; doi:10.1371/journal.pone.0032908)
Supplement: Information S2 — Descriptive information on the study sample. (DOC) [file pone.0032908.s002.doc]

**Supporting information S2 – Descriptive information on the study sample**

Table S1 provides descriptive information on the study participants.

Based on the 2006 Population Census, the mean proportion of residents aged 15 or over with a tertiary education in the residential neighborhood (500 m radius circular areas) was 41%.

| **Table S1**. Descriptive statistics on the participants, RECORD Cohort Study, Paris Metropolitan Area, 2007–2008 (sample excluding participants with missing information for both BMI and WC, n = 7 076). | |
| --- | --- |
| **Variable** | **% n** |
| Proportion of obese participants | 12.4 880 |
| Gender: male | 65.3 4 621 |
| Cohabitation status: living alone | 29.9 2 118 |
| Mother’s education: primary school or less | 45.7 3 232 |
| Individual education: no education | 7.7 543 |
| Employment status: unemployed | 14.9 1 057 |
| Occupation: low white collar worker | 38.5 2 725 |
| Occupation: blue collar worker | 11.0 778 |
| Reporting financial strain | 16.5 1 169 |
| Non-ownership of dwelling | 45.5 3 216 |
| Born in a low human development country | 4.8 339 |
| Abbreviations: BMI, body mass index; WC, waist circumference. | |
