# Supplementary material for: Associations of Supermarket Characteristics with Weight Status and Body Fat: A Multilevel Analysis of Individuals within Supermarkets (RECORD Study)
Source: PLoS One. 2012 Apr 4;7(4):e32908. doi: 10.1371/journal.pone.0032908 (PMC3319546; doi:10.1371/journal.pone.0032908)
Supplement: Information S3 — Information on participants' shopping behavior. (DOC) [file pone.0032908.s003.doc]

**Supporting information S3 – Information on participants’ shopping behavior**

As shown in Table S2 (first column), descriptive data revealed that the proportion of participants who shopped in their own neighborhood tended to increase with socioeconomic status, especially with education of neighborhood residents.

Regarding distance to the supermarket (Table S2, second column), irregular patterns of associations were observed between individual/neighborhood socioeconomic status (individual education, household income, neighborhood proportion of high educated residents, and neighborhood income) and distance to the supermarket. These descriptive data indicated that the distance to the primary supermarket was larger for residents of high income and high education neighborhoods.

Relationships with the proportion of people shopping in their neighborhood and with the average distance to the primary supermarket therefore yielded seemingly contradictory findings. However, the contradiction is only apparent: residents of high educated neighborhoods more frequently shopped in their residential neighborhood (possibly due to more frequently residing in urban centers) but at the same time covered larger *average* distances to their primary supermarket (some of them having holiday homes or working far from their residence).

We then used multilevel regression models to assess which of the individual or neighborhood socioeconomic indicators were more particularly related to the distance to the primary supermarket. For a better understanding of food purchasing behavior (see Table S3), we estimated individual/neighborhood socioeconomic differences in the distance to the primary supermarket before and after adjustment for the type of supermarket where participants shopped (citymarket, hypermarket, small/large supermarket, hard discount supermarket, organic shop).

| **Table S2.** Percentage of participants shopping in their administrative residential neighborhood and distance to the participants’ primary supermarket according to individual/neighborhood socioeconomic status, RECORD Cohort Study, Paris Metropolitan Area, 2007–2008 (sample excluding participants with missing information for both BMI and WC, n = 7 076). | | |
| --- | --- | --- |
| **Variable** | **Percentage shopping in their neighborhood (p-value^a^)** | **Distance in meters (p-value^a^)** |
| Individual education |  |  |
| Low | 9.8% | 2410 |
| Mid-low | 11.0% | 3102 |
| Mid-high | 11.0% | 3326 |
| High | 12.4% (p = 0.050) | 2947 (p < 0.001) |
| Household income |  |  |
| Low | 10.8% | 2608 |
| Mid-low | 10.9% | 3348 |
| Mid-high | 10.9% | 2614 |
| High | 12.8% (p = 0.065) | 3340 (p < 0.001) |
| Neighborhood education |  |  |
| Low | 8.8% | 2988 |
| Mid-low | 10.1% | 3071 |
| Mid-high | 12.2% | 2715 |
| High | 14.4% (p < 0.001) | 3427 (p < 0.001) |
| Neighborhood income |  |  |
| Low | 10.1% | 2820 |
| Mid-low | 11.8% | 3004 |
| Mid-high | 12.0% | 2878 |
| High | 11.5% (p = 0.195) | 3499 (p < 0.001) |
| Abbreviations: BMI, body mass index; WC, waist circumference.  ^a^P-values in the first column were obtained from the Cochran-Armitage trend test, while p-values in the second column were obtained from the Jonckheere-Terpstra test. | | |

| **Table S3.** Association between neighborhood education and distance to the primary supermarket, estimated from multilevel linear models with participants nested within neighborhoods before and after adjustment for the type of supermarket, RECORD Cohort Study, Paris Metropolitan Area, 2007–2008 (sample excluding participants with missing information for both BMI and WC, n = 7 076). | | |
| --- | --- | --- |
|  | **Before adjustment for primary supermarket**  **β (95% CI)** | **After adjustment for primary supermarket**  **β (95% CI)** |
| Neighborhood education (vs. low) |  |  |
| Mid-low | +96 (–833, +1024) | +761 (–178, +1700) |
| Mid-high | –201 (–1142, +740) | +1173 (+185, +2160) |
| High | +380 (–571, +1331) | +2420 (+1392, 3448) |
| Supermarket type (vs. citymarket) |  |  |
| Small supermarket |  | +191 (–897, +1279) |
| Large supermarket |  | +3777 (+2713, +4840) |
| Hypermarket |  | +4859 (+3852, +5865) |
| Hard discount supermarket |  | +2067 (+815, +3318) |
| Organic shop |  | +1914 (–2041, +5868) |
| Abbreviations: BMI, body mass index; CI, confidence interval; WC, waist circumference. | | |

This additional adjusted analysis revealed that the distance to the primary supermarket increased with the educational level of the residential neighborhood (the relationship was of strong magnitude and dose-response) once supermarket type was adjusted for, i.e., among participants using a similar type of supermarket. All other individual/neighborhood socioeconomic indicators were unassociated with the distance to the supermarket after taking into account neighborhood education.

We then compared the effective participants’ primary supermarket to the available supermarkets nearby their residence. Overall, we found that 29.5% of the participants performed most of their food shopping at the closest available supermarket. As shown in Table S4, regression modeling applied to this outcome indicated that participants residing in neighborhoods with a high average education level were more likely to shop at the closest supermarket available from their residence. After taking neighborhood education into account in the model, none of the other individual/neighborhood socioeconomic indicators were associated with shopping or not at the closest available supermarket.

| **Table S4.** Associations between neighborhood education and (i) shopping at the closest available supermarket, (ii) shopping at the closest supermarket of a particular type, and (iii) shopping at the closest supermarket of a particular brand, estimated from multilevel logistic regression models, RECORD Cohort Study, Paris Metropolitan Area, 2007–2008 (n = 7 003) | |
| --- | --- |
|  | **OR (95% CI)** |
| Shopping or not at the closest available supermarket |  |
| Neighborhood education (vs. low) |  |
| Mid-low | 1.16 (0.96, 1.40) |
| Mid-high | 1.48 (1.22, 1.80) |
| High | 1.69 (1.38, 2.07) |
| Shopping or not at the closest available supermarket of a particular type |  |
| Neighborhood education (vs. low) |  |
| Mid-low | 1.26 (1.07, 1.49) |
| Mid-high | 1.39 (1.17, 1.66) |
| High | 1.22 (1.02, 1.47) |
| Shopping or not at the closest available supermarket of a particular brand |  |
| Neighborhood education (vs. low) |  |
| Mid-low | 0.98 (0.81, 1.18) |
| Mid-high | 0.97 (0.79, 1.18) |
| High | 0.85 (0.70, 1.05) |
| Abbreviations: CI, confidence interval; OR, odds ratio. | |
