# Supplementary material for: Associations of Supermarket Characteristics with Weight Status and Body Fat: A Multilevel Analysis of Individuals within Supermarkets (RECORD Study)
Source: PLoS One. 2012 Apr 4;7(4):e32908. doi: 10.1371/journal.pone.0032908 (PMC3319546; doi:10.1371/journal.pone.0032908)
Supplement: Information S4 — Between-neighborhood and between-supermarket variations in BMI and WC from models adjusted for age and gender. (DOC) [file pone.0032908.s004.doc]

**Supporting information S4 – Between-neighborhood and between-supermarket variations in BMI and WC from models adjusted for age and gender**

Table S5 provides components of between-residential neighborhood variability, between-supermarket variability, and residual individual-level variability estimated from models for BMI and WC.

| **Table S5.** Classical and cross-classified multilevel linear models for BMI and WC adjusted for age and sex, RECORD Cohort Study, Paris Metropolitan Area, 2007–2008. | | |
| --- | --- | --- |
|  | **BMI** | **WC** |
| Model 1 |  |  |
| Neighborhood variance (95% CI) | 0.97 (0.69, 1.45) | 4.90 (3.21, 8.37) |
| Individual-level variance (95% CI) | 15.97 (15.39, 16.59) | 112.72 (108.6, 117.1) |
| % of variance at the neighborhood level | 5.7% | 4.2% |
| Akaike Information Criterion | 40 068.3 | 52 673.9 |
|  |  |  |
| Model 2 |  |  |
| Supermarket variance (95% CI) | 0.55 (0.37, 0.93) | 3.12 (1.94, 5.85) |
| Individual-level variance (95% CI) | 16.41 (15.86, 16.99) | 114.7 (110.8, 118.8) |
| % of variance at the supermarket level | 3.3% | 2.7% |
| Akaike Information Criterion | 40 067.5 | 52 668.3 |
|  |  |  |
| Model 3 |  |  |
| Neighborhood variance (95% CI) | 0.70 (0.45, 1.24) | 3.63 (2.10, 7.70) |
| Supermarket variance (95% CI) | 0.42 (0.26, 0.84) | 2.57 (1.48, 5.53) |
| Individual-level variance (95% CI) | 15.81 (15.23, 16.43) | 111.47 (107.32, 115.87) |
| % of variance at the neighborhood level | 4.2% | 3.1% |
| % of variance at the supermarket level | 2.5% | 2.2% |
| Akaike Information Criterion | 40 050.1 | 52 658.9 |
| Abbreviations: BMI, body mass index; CI, confidence interval; WC, waist circumference. | | |

Table S5 successively reports estimates from models including a random effect (i) only at the residential neighborhood level; (ii) only at the supermarket level; and (iii) both at the residential neighborhood and supermarket levels. It also provides the Akaike Information Criterion for each of these models.

As stated in the main text, the Akaike Information Criterion was lower in the model comprising both the residential neighborhood-level and the supermarket-level random effects.
