# Supplementary material for: Associations of Supermarket Characteristics with Weight Status and Body Fat: A Multilevel Analysis of Individuals within Supermarkets (RECORD Study)
Source: PLoS One. 2012 Apr 4;7(4):e32908. doi: 10.1371/journal.pone.0032908 (PMC3319546; doi:10.1371/journal.pone.0032908)
Supplement: Information S5 — Models with individual and residential neighborhood variables associated with BMI and WC. (DOC) [file pone.0032908.s005.doc]

**Supporting information S5 – Models with individual and residential neighborhood variables associated with BMI and WC**

| **Table S6.** Associations between, on the one hand, individual sociodemographic variables and neighborhood education and, on the other hand, BMI and WC, estimated from cross-classified multilevel linear models (all effects in the same column are adjusted for each other), RECORD Cohort Study, Paris Metropolitan Area, 2007–2008. | | | | |
| --- | --- | --- | --- | --- |
|  | **Δ BMI in kg/m²** | **95% CI** | **Δ WC in cm** | **95% CI** |
| Age (1-year increase) | +0.16 +0.09, +0.24 | | +0.50 +0.28, +0.73 | |
| Age squared | –0.00 –0.00, –0.00 | | –0.00 –0.01, –0.00 | |
| Male (vs. female) | +1.09 +0.88, +1.30 | | +11.78 +11.20, +12.33 | |
| Living alone (vs. cohabiting) | –0.33 –0.54, –0.11 | | –1.00 –1.57, –0.42 | |
| Mother’s education (vs. tertiary school) |  | |  | |
| Secondary school | +0.26 –0.04, +0.55 | |  | |
| Primary school or less | +0.33 +0.01, +0.64 | |  | |
| Individual education (vs. high) |  | |  | |
| Mid-high | +0.21 –0.05, +0.46 | | +0.24 –0.43, +0.91 | |
| Mid-low | +0.78 +0.48, +1.08 | | +1.59 +0.79, +2.38 | |
| Low | +1.36 +0.92, +1.79 | | +2.72 +1.57, +3.86 | |
| Employment status (vs. employed) |  | |  | |
| Unemployed | –0.41 –0.70, –0.13 | | –0.88 –1.64, –0.12 | |
| Retired | +0.19 –0.22, +0.59 | | +0.30 –0.81, +1.42 | |
| Occupation (vs. high white collar  worker) |  | |  | |
| Intermediate occupation | –0.34 –0.78, +0.10 | | –1.25 –2.43, –0.07 | |
| Low white collar worker | –0.23 –0.49, +0.03 | | –0.51 –1.20, +0.18 | |
| Blue collar worker | –0.80 –1.19, –0.41 | | –1.81 –2.85, –0.78 | |
| Financial strain | +0.65 +0.36, +0.93 | | +2.10 +1.36, +2.84 | |
| Non-ownership of dwelling | +0.54 +0.32, +0.76 | | +1.50 +0.91, +2.08 | |
| Human development of country of birth  (vs. mainland France) |  | |  | |
| High level | +0.16 –0.18, +0.49 | |  | |
| Intermediate level | +0.40 +0.12, +0.68 | |  | |
| Low level | +0.83 +0.35, +1.30 | |  | |
| Neighborhood education (vs. high) |  | |  | |
| Mid-high | +0.20 –0.08, +0.47 | | +0.37 –0.37, +1.11 | |
| Mid-low | +0.39 +0.11, +0.68 | | +0.70 –0.06, +1.47 | |
| Low | +1.35 +1.04, +1.65 | | +3.14 +2.33, +3.95 | |
| Abbreviations: BMI, body mass index; CI, confidence interval; WC, waist circumference. | | | | |

Before adding the supermarket-level variables, we constructed models for BMI and WC retaining all of the individual and residential neighborhood variables that were associated with each outcome. These models included a random effect both at the residential neighborhood level and at the supermarket level.

As shown in Table S6, slightly different individual-level variables were retained in the models for BMI and WC. Education (rather than income or dwelling values) was the socioeconomic characteristic of residential neighborhoods most strongly associated with BMI or WC.
