# Supplementary material for: Associations of Supermarket Characteristics with Weight Status and Body Fat: A Multilevel Analysis of Individuals within Supermarkets (RECORD Study)
Source: PLoS One. 2012 Apr 4;7(4):e32908. doi: 10.1371/journal.pone.0032908 (PMC3319546; doi:10.1371/journal.pone.0032908)

**Supporting information S6 – Identification of the residential and supermarket neighborhood socioeconomic variables most strongly associated with BMI or WC and assessment of the optimal spatial scale of measurement of the variables**

Once the individual sociodemographic variables associated with each outcome were selected, we examined which of the 3 socioeconomic indicators described in the main text (proportion of high educated residents, median income, and mean value of dwellings) was most strongly associated with BMI or WC. Moreover, we assessed the spatial scale on which each socioeconomic contextual variable was most strongly associated with BMI and WC.

Model selection was performed on the basis of models containing the individual variables associated with each outcome, and both a residential neighborhood random effect and a supermarket level random effect. In this sensitivity analysis, we compared circular areas with the following radiuses: 100 m, 250 m, 500 m, 750 m, 1 000 m, 2 500 m, 5 000 m, 7 500 m, and 10 000 m. As noted in the main text, the socioeconomic status of supermarket catchment areas was also determined within circular areas with a varying radius corresponding to the 75^th^ percentile of the straight-line distance from home to the supermarkets of each particular brand observed in our sample. The brand-specific radiuses are reported for information in Table S7.

We compared models including each socioeconomic contextual variable, for the residential neighborhood and for the supermarket neighborhood, measured on each spatial scale (fixed scales and brand-specific scale). Each socioeconomic variable was introduced as a continuous variable in a separate model incorporating only the individual sociodemographic factors.

We used the Akaike Information Criterion to select the model with the best overall combination of fit to the data and parsimony. Our *a priori* decision was to retain, as a residential neighborhood socioeconomic indicator, and as a supermarket neighborhood socioeconomic indicator, the socioeconomic variable and the spatial scale of measurement leading to the lowest value of the Akaike Information Criterion.

| **Table S7.** Brand-specific radiuses used in the definition of circular supermarket catchment areas (corresponding to the 75^th^ percentile of straight-line distance from home to supermarkets of each brand in the sample), RECORD Cohort Study, Paris Metropolitan Area, 2007–2008. | |
| --- | --- |
| **Brand name** | **Brand-specific radius (m)** |
| 8 à Huit | 944 |
| Aldi | 2 810 |
| Auchan | 3 951 |
| Biocoop | 2 469 |
| Carrefour | 3 818 |
| Casino | 734 |
| Champion | 1 115 |
| Cocci Market | 3 795 |
| Cora | 2 051 |
| Corsaire | 1 560 |
| Ed | 941 |
| Franprix | 654 |
| G20 | 1 438 |
| Géant Casino | 1 103 |
| Hyper U | 23 391 |
| Inno | 1 057 |
| Intermarché | 1 153 |
| Leader Price | 1 882 |
| Leclerc | 2 555 |
| Les Nouveaux Robinson | 2 690 |
| Lidl | 2 865 |
| Marché U | 907 |
| Metro | 3 319 |
| Monoprix | 791 |
| Naturalia | 569 |
| Shopi | 588 |
| Simply Market | 964 |
| Super U | 1 729 |
|  | |

Figures S1A and S1B provide the Akaike Information Criterion of the different models estimated. Regarding residential neighborhood characteristics, we reported in a previous publication [1] that education was a clearly better predictor of BMI or WC than median income or dwelling values. Accordingly, in Figures S1A and S1B, for the sake of clarity, we only report the sensitivity analysis for the spatial scale of measurement of residential neighborhood education. The fit of the model to the data was the best when residential neighborhood education was measured in 500 m radius circular areas, for both BMI and WC.

Regarding supermarket catchment area characteristics, again, the percentage of high educated residents led to a clearly better fit to the data than the other two socioeconomic variables. Interestingly, Figures S1A and S1B demonstrate that socioeconomic characteristics of supermarket catchment areas were associated with BMI or WC on a much broader scale than residential neighborhood education. In a remarkably consistent way, for all 3 socioeconomic characteristics (education, income, dwelling values) and for both outcomes (BMI and WC), the fit of the model to the data was the best when supermarket neighborhood socioeconomic indicators were assessed within 5 000 m radius circular areas centered on the supermarket. Both for BMI and WC, considering brand-specific radiuses for supermarket catchment areas led to a fit to the data that was comparable to that attained when using 5 000 m radius circular areas. Model fit was slightly better for BMI when using brand-specific radius areas, and slightly better for WC when using 5 000 m radius areas.

Following our *a priori* decision to select the spatial scale of measurement based on the Akaike Information Criterion, all of the adjusted analyses relied on residential neighborhood education measured within 500 m radius circular areas and on supermarket neighborhood education measured within brand-specific radius areas for BMI and 5 000 m radius circular areas for WC.

**References**

1. Leal C, Bean K, Thomas F, Chaix B (2011) Are associations between neighborhood socioeconomic characteristics and body mass index or waist circumference based on model extrapolations? Epidemiology 22: 694-703.

**Figure S1. Akaike Information Criteria from models for BMI (part A) or WC (part B).**

Supermarket neighborhood education, income, or dwelling values or residential neighborhood education measured on different spatial scales were included in separate models, RECORD Cohort Study, Paris Metropolitan Area, 2007–2008.


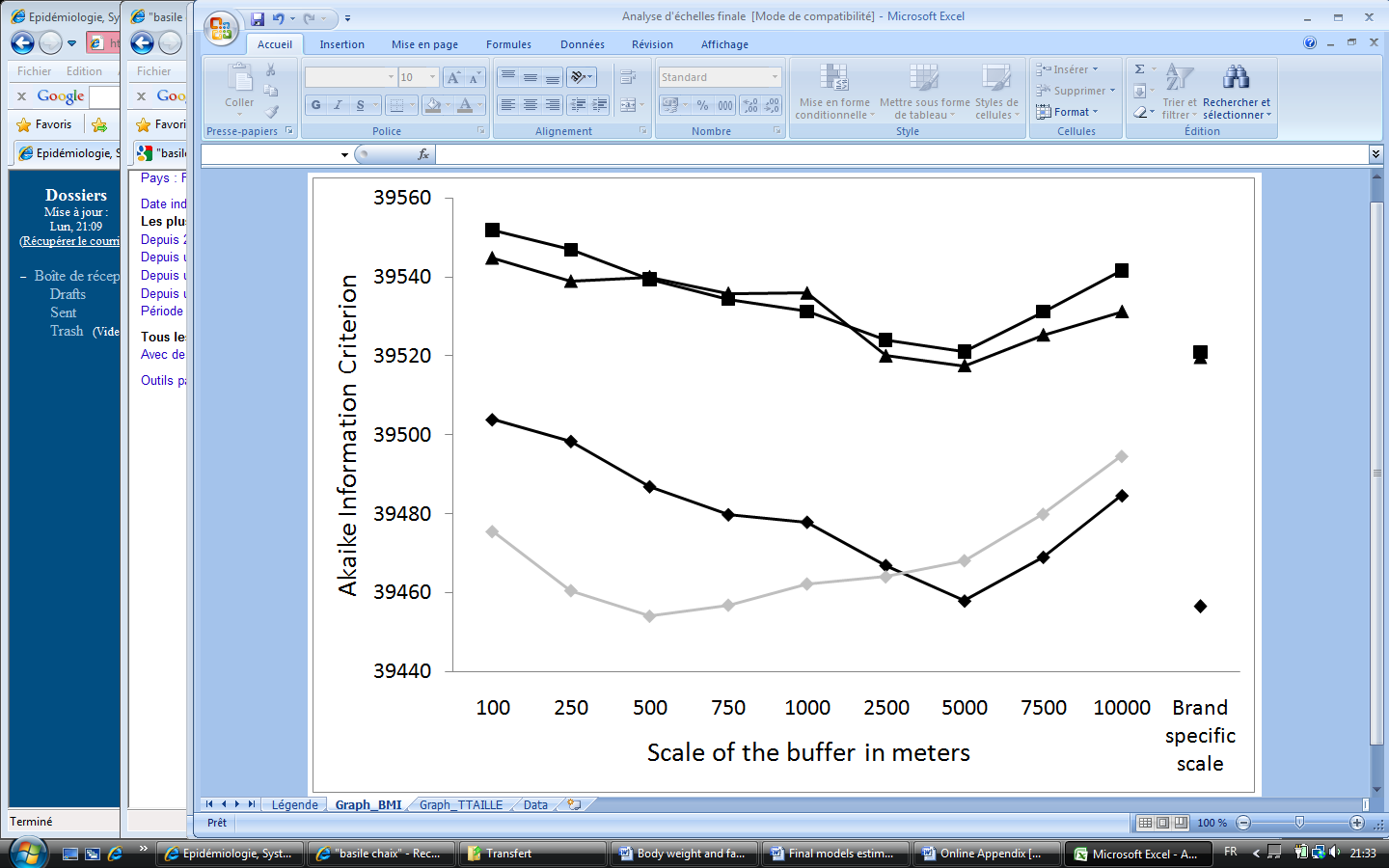
*Part A*


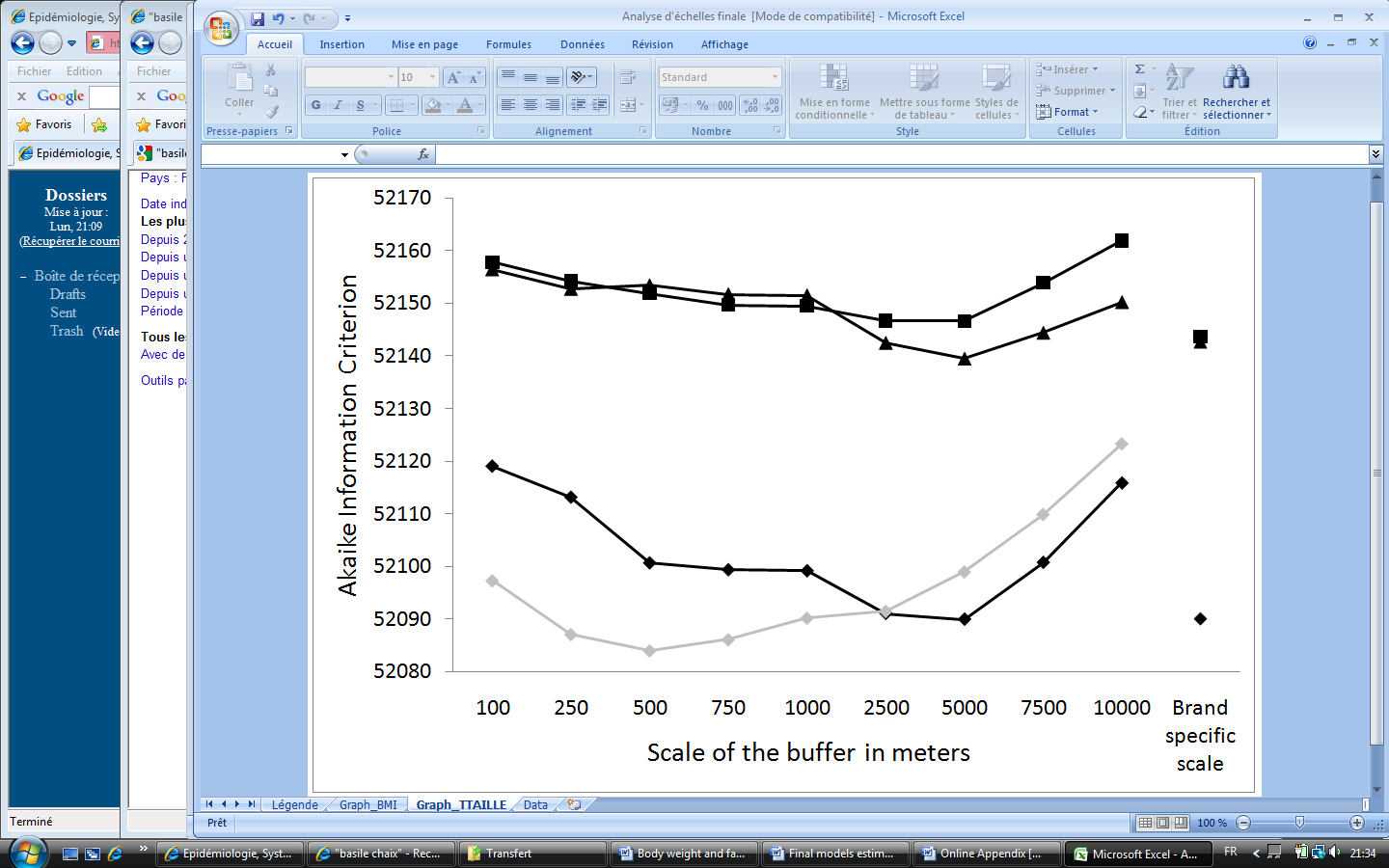
*Part B*


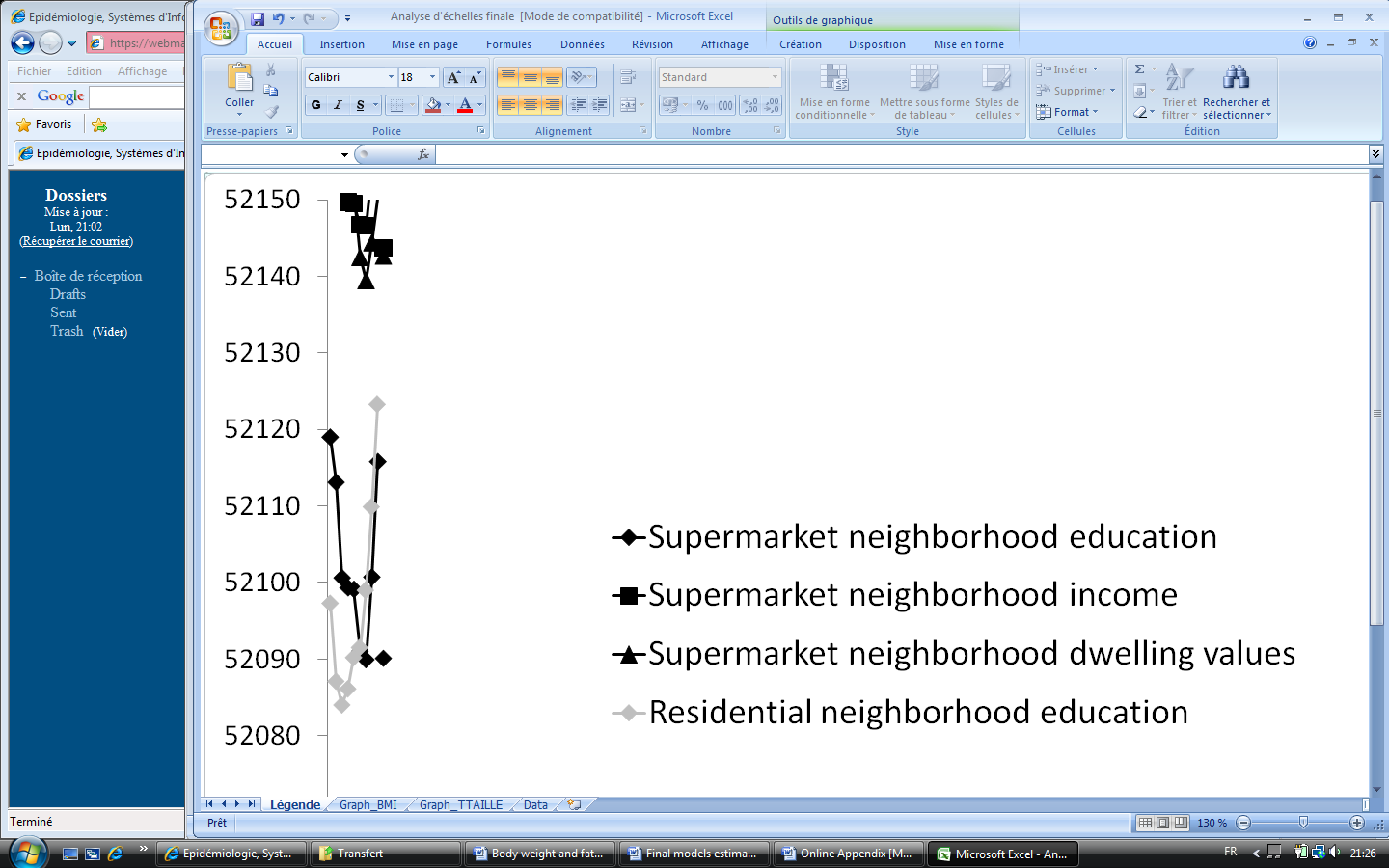

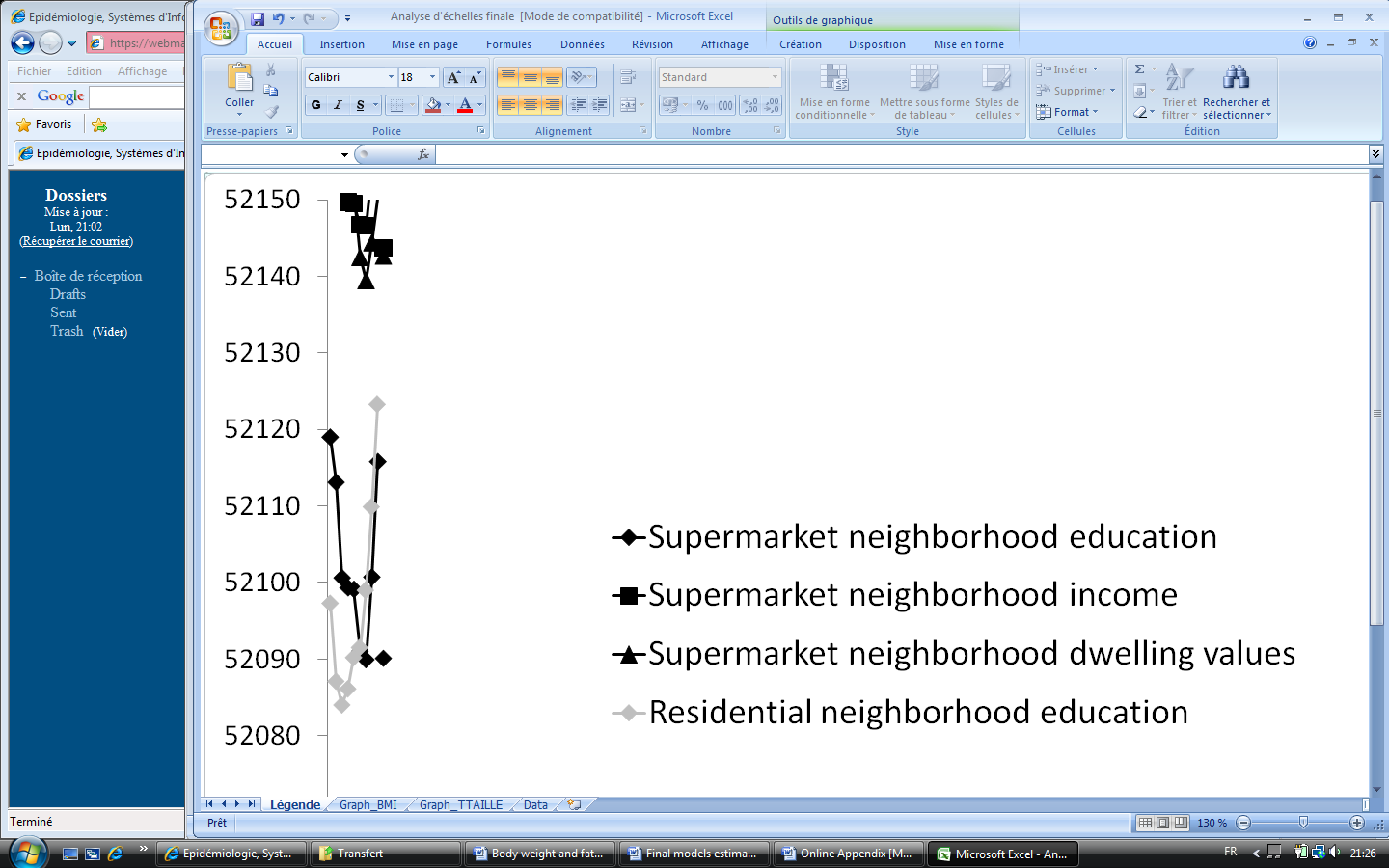

Supplement: Information S6 — Identification of the residential and supermarket neighborhood socioeconomic variables most strongly associated with BMI or WC and assessment of the optimal spatial scale of measurement of the variables. (DOC) [file pone.0032908.s006.doc]
