# Supplementary material for: Associations of Supermarket Characteristics with Weight Status and Body Fat: A Multilevel Analysis of Individuals within Supermarkets (RECORD Study)
Source: PLoS One. 2012 Apr 4;7(4):e32908. doi: 10.1371/journal.pone.0032908 (PMC3319546; doi:10.1371/journal.pone.0032908)
Supplement: Information S7 — Relationships between supermarket type and body mass index (BMI) or waist circumference (WC) estimated through propensity score matching. (DOC) [file pone.0032908.s007.doc]

**Supporting information S7 – Relationships between supermarket type and body mass index (BMI) or waist circumference (WC) estimated through propensity score matching**

In the main article, we control for individual socioeconomic characteristics when estimating associations between supermarket type and BMI/WC. In order to disentangle the “effect” of, e.g., shopping in a hard discount supermarket from that of individual socioeconomic characteristics, we need to have both advantaged and disadvantaged people shopping in hard discount supermarkets and advantaged and disadvantaged people shopping in other supermarkets. In a scenario in which all disadvantaged people would shop in hard discount supermarkets and in which all advantaged people would shop in other supermarkets, it would not be possible to separate the effect of shopping in a specific supermarket from the effects of individual socioeconomic variables on BMI/WC. In order to separate the effects, participants shopping in the different types of supermarkets need to be exchangeable according to their individual socioeconomic characteristics.

We followed a propensity score matching approach described in the following paragraphs to assess whether it is possible to adjust “effects” of supermarket type for individual socioeconomic characteristics without excessive extrapolations of regression models.

Step 1: In 3 separate analyses, we computed individuals’ propensity to use as their primary source of food (i) a small/large supermarket rather than a citymarket; (ii) a hypermarket rather than a citymarket; and (iii) a hard discount supermarket rather than a citymarket. Such propensities were determined as the predicted probability to use the corresponding supermarket type estimated from a multilevel logistic model with individual socioeconomic variables and neighborhood socioeconomic status as the explanatory variables. In these propensity score models, we only retained the individual/neighborhood sociodemographic variables that were associated with shopping in each supermarket type (small/large supermarket, hypermarket, or hard discount supermarket rather than citymarket).

Step 2: In 3 separate analyses, (i) we matched participants shopping in a small/large supermarket with participants shopping in a citymarket according to their propensity to shop in a small/large supermarket rather than in a citymarket (estimated in step 1); (ii) we matched participants shopping in a hypermarket with participants shopping in a citymarket according to their propensity to shop in a hypermarket rather than in a citymarket; and (iii) we matched participants shopping in a hard discount supermarket with participants shopping in a citymarket according to their propensity to shop in a hard discount supermarket rather than in a citymarket. In these 3 cases, in order to account for the different sample size shopping at each supermarket type, we performed 3:1 matching, 3:1 matching, and 1:2 matching, respectively. Of particular interest for us, we examined the extent to which each of the samples was reduced after matching, a large reduction in sample size indicating a limited exchangeability of participants shopping in the different supermarket types. For BMI and WC respectively, sample size decreased by 33% / 33% for the samples allowing us to compare small/large supermarkets with citymarkets, by 64% / 64% for the samples allowing us to compare hypermarkets with citymarkets, and by 63% / 64% for the samples allowing us to compare hard discount supermarkets with citymarkets. The sharp decrease in sample size after matching observed for the samples allowing us to compare citymarkets with hypermarmets or hard discount supermarkets suggests that participants shopping in the former and in the latter are exchangeable to a limited extent according to their individual socioeconomic profile.

Step 3: We reestimated the associations between supermarket type (small/large supermarket vs. citymarket; hypermarket vs. citymarket; and hard discount supermarket vs. citymarket) and BMI or WC in each of the 3 samples generated through propensity score matching, in which the exchangeability of participants shopping in different supermarket types according to individual socioeconomic characteristics is ensured.

Associations between supermarket type and BMI/WC before and after matching are reported in Table 5 of the main article. After matching, the association with shopping in a small/large supermarket rather than in a citymarket was only retrieved for WC. Shopping in a hypermarket remained associated with increased BMI and WC after matching. However, the associations observed before matching between shopping in a hard discount supermarket and increased BMI and WC were not retrieved after matching, as a likely consequence of the initial small sample size and of the sharp decrease in sample size after matching (that reflects, as noted above, the limited exchangeability according to individual socioeconomic status of participants shopping in citymarkets and hard discount supermarkets). It suggests that the adjusted associations between shopping in a hard discount supermarket and BMI or WC were to some extent based on model extrapolations when observed in the whole sample and could not be documented in samples restricted to exposed and unexposed participants exchangeable according to their individual socioeconomic characteristics.
